# Significance of coagulation and fibrinolysis markers for benign and malignant soft tissue tumors

**DOI:** 10.1186/s12885-021-08091-1

**Published:** 2021-04-07

**Authors:** Kunihiro Asanuma, Tomoki Nakamura, Tomohito Hagi, Takayuki Okamoto, Kouji Kita, Koichi Nakamura, Yumi Matsuyama, Keisuke Yoshida, Yumiko Asanuma, Akihiro Sudo

**Affiliations:** 1 Department of Orthopedic Surgery, Mie University School of Medicine, 2-174 Edobashi, Tsu City, Mie 514-8507 Japan; 2 Department of Pharmacology, Faculty of Medicine, Shimane University, Izumo, Shimane Japan

**Keywords:** Soft tissue sarcoma, Metastasis, Prognosis, Coagulation, Fibrinolysis, D-dimer, Plasmin-α2 plasmin inhibitor complex, Soluble fibrin, And thrombin-antithrombin III complex

## Abstract

**Background:**

The intimate relationship between coagulation and fibrinolysis in malignant tumors is a well-known phenomena, with the malignant phenotype enhancing coagulation and fibrinolysis. We hypothesized that soft tissue sarcoma (STS) affects the expression of coagulation and fibrinolysis markers, which could be used to distinguish STS from benign soft tissue tumors. We analyzed the correlations between plasma levels of D-dimer (DD), plasmin-α2 plasmin inhibitor complex (PIC), soluble fibrin (SF), and thrombin-antithrombin III complex (TAT) in benign soft tissue tumors and STS to elucidate whether these markers can be used to predict STS.

**Methods:**

Plasma DD, PIC, SF and TAT levels in primary soft tissue tumors (benign 67, STS 68) were measured before biopsy or treatment. The marker levels were analyzed and compared to various clinicopathological parameters.

**Results:**

In malignancy (STS), the average DD, PIC and SF levels were significantly higher than in benign tumors. Multivariate logistic analysis of continuous variables indicated that only PIC exhibited a significant difference (OR: 24.5, 95%CI: 3.55–170, *p* = 0.0012). Receiver operating characteristic curve analysis produced area under the curve values for DD: 0.691, PIC: 0.784, SF: 0.734 and TAT: 0.588. Youden’s index was used to establish thresholds of 0.37 (DD), 0.80 (PIC), 0.90 (SF) and 0.82 (TAT). Threshold values for PIC and SF indicated high specificity (0.881, 0.791) and high positive predictive value (0.818, 0.745), respectively. The highest accuracy value among the markers was observed for PIC (0.704). Significant differences in multivariate analysis of binary variables were demonstrated by categorizing low and high groups based on their threshold, PIC (≥0.80) (OR: 3.36, 95%CI: 1.19–9.43, *p* = 0.0212) and SF (≥0.90) (OR: 2.63, 95%CI: 1.04–6.66, *p* = 0.0404) .

**Conclusions:**

Of the coagulation and fibrinolysis markers studied, increased PIC levels were related to STS and over 0.80 PIC was the most suitable for the prediction of STS, which, along with other diagnostic tools, represents a helpful subsidiary tool for the prediction of STS.

## Background

It is well-known that malignant tumors are highly correlated with coagulation and fibrinolysis. Many tumor cells express tissue factor (TF) and urokinase-type plasminogen activator receptor (uPAR) on their surface. TF is a coagulation factor that activates the extrinsic coagulation cascade, thereby leading to the generation of thrombin and fibrin. Thrombin stimulates tumor cell adhesion to platelets, endothelial cells, extracellular matrix proteins, as well as tumor cell mitogenesis [1, 2]. Previous reports of breast cancer showed that thrombin increased invasive activity through the thrombin receptor and that inhibition of protease activated receptor-1 (PAR-1) expression reduced invasive potential [3, 4]. Thus, the effects of thrombin may account for the observation that thrombin-pretreated melanoma cells markedly enhance pulmonary metastasis in vivo [1]. In contrast, uPAR, a main fibrinolytic factor of cancer cells, forms a complex with urokinase-type plasminogen activator (uPA) and converts plasminogen into plasmin. The generated plasmin induces a variety of proteolyses, including fibrin degradation. Moreover, plasmin not only cleaves extracellular matrices but also activates pro-MMPs [5]. Overexpression of uPAR in breast cancer enhanced tumor invasion, growth and metastasis [6]. Additionally, breast cancer patients with high expression of uPA and plasminogen activator inhibitor-1 (PAI-1) in tumor tissues had lower survival than the low expression group [7]. Thus, the malignant phenotype appears to enhance coagulation and fibrinolysis.

The state of coagulation and fibrinolysis can be evaluated by examining several markers such as thrombin-antithrombin III complex (TAT), soluble fibrin (SF), plasmin and alpha 2 plasmin inhibitor complex (PIC) and D-dimer (DD). These markers were examined in this study. After thrombin is generated by activation of the coagulation cascade, part of it forms a complex with antithrombin III (TAT) [1]. Some thrombin cleaves fibrinogen and the resultant fibrin monomers can form a complex with fibrinogen (SF) [2]. Furthermore, fibrinolysis can be evaluated by assessing fibrinolysis markers such as plasmin and alpha 2 plasmin inhibitor complex (PIC) or D-dimer. After plasmin is generated from plasminogen, it can form a complex with alpha 2 plasmin inhibitor [3]. DD consists of two cross-linked D fragments that generated by degradation of fibrin following proteolysis by plasmin [4], and can be used as an indication of plasmin-induced fibrinolysis after fibrin formation [5].

Thus, we hypothesized that soft tissue sarcoma (STS) could affect the expression of coagulation and fibrinolysis markers, which could be used to distinguish STS from benign soft tissue tumors. To examine the predictive value of markers of coagulation or fibrinolysis for discriminating between benign soft tissue tumors and STS, we analyzed the correlation between plasma levels of TAT, SF, PIC or DD and clinical parameters in soft tissue tumor patients.

## Methods

### Patients

A total of 135 patients (76 men and 59 women) with primary soft tissue tumors who visited Mie University Hospital from 2012 to 2014 were enrolled in this study. Patients who had local recurrence, were referred for additional resection after inadequate resection in a previous hospital, or had trauma, surgical treatment, thrombosis and disseminated intravascular coagulation (DIC) were excluded from this study. Histopathological diagnoses based on the AJCC (American Joint Committee on Cancer) system were verified by independent pathologists. Blood samples from all patients were obtained (stored in sodium citrate tubes) before biopsy or treatment.

The levels of D-dimer (DD: μg/ml), plasmin-α2 plasmin inhibitor complex (PIC: μg/ml), soluble fibrin (SF: μg/ml) or thrombin-antithrombin III complex (TAT: ng/ml) in plasma were quantified using a chemiluminescent enzyme immunoassay or latex photometric immunoassay. Written, informed consent was obtained from each patient. For patients below the age 19 years, informed consent was obtained from their parents or legal guardian. This study was approved by the Ethics Committee of the Mie University Graduate School of Medicine (approval number: 1310). All procedures performed in studies involving human participants were in accordance with the ethical standards of the Ethics Committee of Mie University and with the Declaration of Helsinki.

### Statistical analysis

Clinicopathological analysis was performed to compare the plasma levels of TAT, SF, PIC or DD to various clinical parameters using the Mann-Whitney test or Kruskal Wallis test for quantitative data and Fisher’s exact test for qualitative data. To evaluate the threshold for detecting STS, receiver operating characteristic (ROC) curves were generated. ROC curves were created by plotting sensitivity on the y-axis and the false positive rate (1-specificity) on the x-axis. The area under the curve (AUC) of the ROC curves was assessed to measure the effectiveness of TAT, SF, PIC or DD levels as markers of malignancy. Univariate and multivariate logistic regression analyses were used to screen and estimate risk factors for malignancy of soft tissue tumors. *P* < 0.05 was considered significant. The EZR software program was used for statistical analyses [6].

## Results

### Patient and tumor characteristics

The average age of the patients was 55.9 years (range: 12–92 years), and the median tumor size was 10.3 cm (range: 1–31 cm). The distribution between benign and STS for patients ≥60 years of age and patients with tumor size ≥10 cm were significantly different (Table 1).

**Table 1:** Patient characteristics in benign soft tissue tumors and STS

Characteristics		n	Benign	STS	***p***-value
Sex	Male	76	40	36	0.489
	Female	59	27	32	
Age	< 60	75	49	26	**0.0000603**
	≥60	60	18	42	
Tumor size	< 10	78	48	30	**0.00165**
	≥10	57	19	38	
Location	Extremity	68	39	29	0.0859
	Trunk	67	28	39	
Tumor depth	Superficial	51	27	24	0.597
	Deep	84	40	44	

Sex, age, tumor size, location, and tumor depth for benign and STS patients are enumerated. Distributions were compared for each parameter using Fisher’s exact test

Histopathological diagnoses were as follows: 67 benign tumors were made up of 26 lipomas, 13 schwannomas, 7 fibromatosis, 5 chronic expanding hematomas, 3 neurofibromas, 3 pigmented villonodular synovitis (PVNS), and 10 others; while the 68 STS were made up of 29 liposarcomas (17 well-differentiated liposarcomas, 6 dedifferentiated liposarcomas, and 6 myxoid liposarcomas), 13 undifferentiated pleomorphic sarcomas, 9 myxofibrosarcomas, 4 leiomyosarcomas, 3 synovial sarcomas, 3 malignant peripheral nerve sheath tumors, and 7 others.

### DD, PIC, SF and TAT levels

**Fig. 1:**
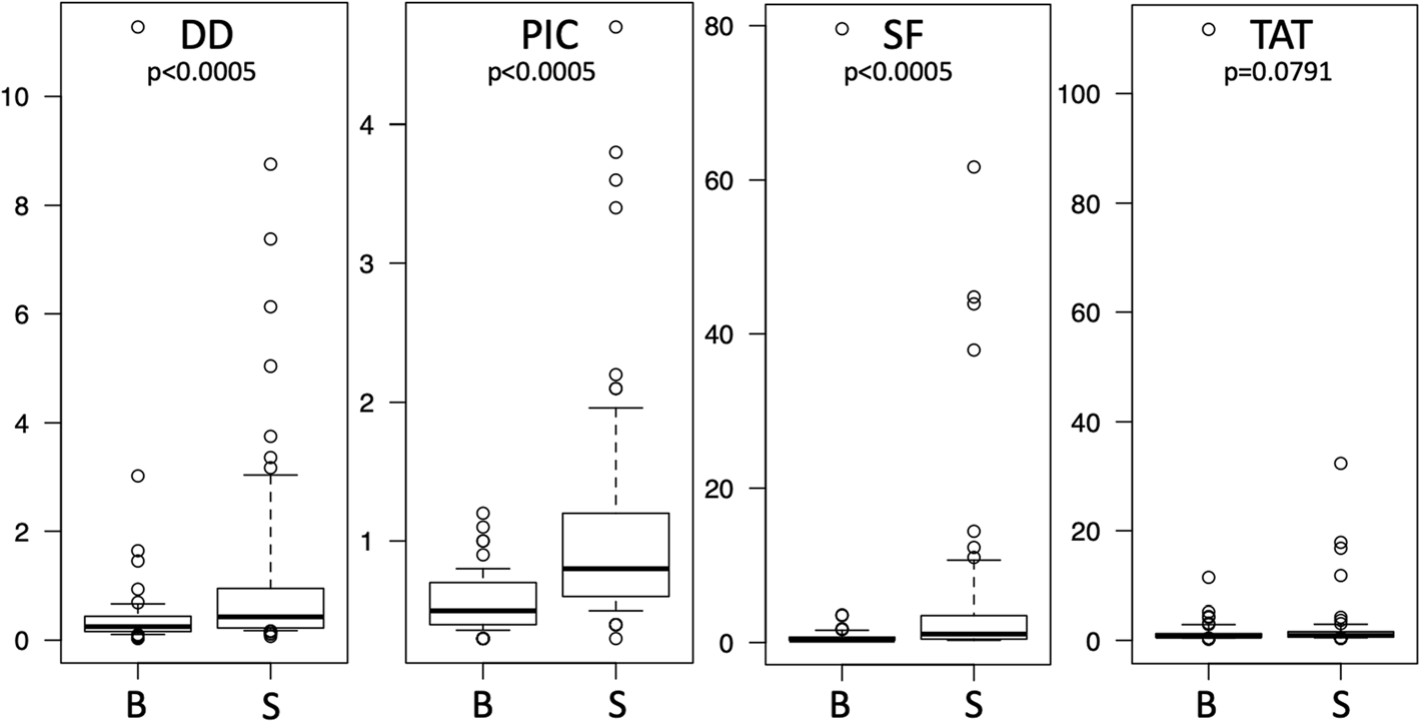
Evaluation of DD, PIC, SF and TAT in patients with benign tumors or STS. DD, PIC, and SF levels were significantly higher in STS (S) than in benign (B) tumors (Mann-Whitney test)

DD, PIC, SF, and TAT levels were compared based on clinical characteristics. Sex, tumor location and tumor depth were not significantly different according to DD, PIC, SF, and TAT levels. Patients with age ≥ 60 years showed significantly elevated levels of DD, PIC, SF and TAT. Patients with tumor size ≥10 cm showed significantly higher levels of DD, PIC and SF (Table 2). In the malignancy group (STS), average DD, PIC and SF levels were significantly higher than in the benign tumor group, according to the Mann-Whitney test (Fig. 1). In particular, chronic expanding hematoma and PVNS have increased risk for alterations in thrombosis or fibrinolysis status. DD, PIC, SF, and TAT levels in chronic expanding hematoma were 3.13 ± 4.57, 0.62 ± 0.29, 1.20 ± 1.33 and 3.26 ± 4.59, respectively. Only DD showed a significantly higher level than the other benign tumors (*P* = 0.000514, Mann-Whitney test) or STS (*P* = 0.0433, Mann-Whitney test). As chronic expanding hematoma growth is a long-term phenomenon, reactions in hematoma probably progress to the final fibrin degradation product (DD). High DD is useful for diagnosing chronic expanding hematoma in benign tumors. DD, PIC, SF, and TAT levels in PVNS were 0.33 ± 0.29, 0.53 ± 0.23, 0.43 ± 0.23, and 0.69 ± 0.19, respectively. PVNS did not exhibit significant differences in DD, PIC, SF, or TAT relative to other benign tumors.

**Table 2:** Average values of DD, PIC, SF and TAT according to patient characteristics

		DD (SD)	PIC (SD)	SF (SD)	TAT (SD)
Sex	Male	0.90 (1.81)	0.80 (0.73)	3.51 (11.2)	3.03 (12.8)
	Female	0.72 (1.24)	0.83 (0.58)	3.40 (9.80)	1.96 (4.76)
Age	< 60	*0.39 (0.86)	*0.59 (0.51)	*0.84 (1.43)	*0.99 (0.89)
	≥60	*1.35 (2.05)	*1.09 (0.74)	*6.74 (15.2)	*4.53 (15.0)
Tumor size	< 10	*0.64 (1.54)	*0.72 (0.70)	*1.30 (2.15)	1.30 (1.56)
	≥10	*1.08 (1.62)	*0.94 (0.59)	*6.43 (15.6)	4.29 (15.4)
Location	Extremity	0.85 (1.77)	0.74 (0.49)	2.51 (7.03)	1.45 (1.99)
	Trunk	0.79 (1.37)	0.89 (0.80)	4.44 (13.2)	3.70 (14.2)
Tumor depth	Superficial	0.80 (1.86)	0.83 (0.75)	3.53 (10.4)	1.88 (3.11)
	Deep	0.83 (1.39)	0.80 (0.61)	3.43 (10.7)	2.98 (12.6)
Malignancy	Benign	*0.52 (1.40)	*0.55 (0.19)	*1.85 (9.66)	2.91 (13.5)
	STS	*1.11 (1.70)	*1.08 (0.84)	*5.06 (11.2)	2.23 (4.84)

Average values of DD, PIC, SF and TAT are shown for each parameter. * indicates significant difference by the Mann-Whitney test (*P* < 0.05)

### Continuous variable logistic regression analysis of DD, PIC, SF and TAT

Logistic regression analyses were performed to elucidate the association of multiple factors in the detection of malignancy. Univariate analysis indicated that age (≥ 60 years old) (OR: 4.40, 95%CI: 2.12–9.11, *p* < 0.0005), tumor size (≥ 10 cm) (OR: 3.20, 95%CI: 1.57–6.54, *p* = 0.00143) and PIC (OR: 50.9, 95%CI: 8.70–297, *p* < 0.0001) were significant factors. These factors were utilized in the subsequent multivariate analysis. Of the three factors, only PIC was determined to be a significant predictor of malignancy (OR: 24.5, 95%CI: 3.55–170, *p* = 0.0012). Thus, increases in PIC appear to be associated with significantly increased risk of STS (Table 3).

**Table 3:** Logistic analysis of continuous variables

Univariate logistic analysis				Multiple logistic analysis			
Factor	OR	95%CI	*p*-value	Factor	OR	95%CI	*p*-value
Sex (male)	0.75	0.38–1.50	0.429	Age (≥60)	1.62	0.67–3.88	0.282
Age (≥60)	4.40	2.12–9.11	**0.0005>**	Tumor size (≥10)	1.89	0.82–4.29	0.130
Tumor size (≥10)	3.20	1.57–6.54	**0.00143**	PIC	24.5	3.55–170	**0.0012**
Location (trunk)	1.87	0.94–3.71	0.0717				
Deep	1.25	0.61–1.54	0.549				
DD	1.40	0.99–1.97	0.0565				
PIC	50.9	8.70–297	**0.0001>**				
SF	1.04	0.98–1.10	0.132				
TAT	0.99	0.95–1.03	0.703				

Logistic analysis was performed to detect malignancy using continuous values of DD, PIC, SF and TAT. Multivariate analysis was performed on the significant factors identified in the univariate analysis

### Establishing threshold values and evaluating diagnostic accuracy

**Fig. 2:**
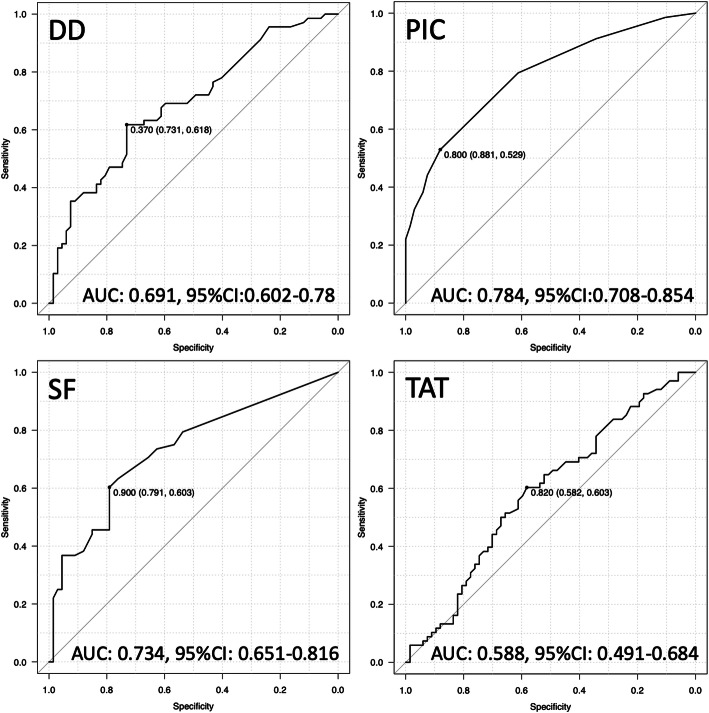
ROC analysis. Thresholds values were determined by based on the Youden’s index. Thresholds for DD, PIC, SF and TAT were 0.37, 0.80, 0.90 and 0.82, respectively. Youden’s index values are presented as threshold (specificity, sensitivity)

Diagnostic accuracy for distinguishing between benign or malignant tumors was evaluated using the area under the ROC curve (AUC). Accordingly, the AUC values for PIC (0.784) and SF (0.734) were higher than for DD (0.691) and TAT (0.588) (Fig. 2). Thresholds for identifying malignant patients using DD, PIC, SF and TAT, derived from the AUC analysis using Youden’s index, were determined as 0.37, 0.80, 0.90 and 0.82, respectively (Fig. 2). Threshold values for PIC and SF indicated high specificity (0.881, 0.791) and high positive predictive value (0.818, 0.745), respectively (Fig. 2, Table 4). A high degree of diagnostic accuracy for STS was observed for PIC (over 0.80) and SF (over 0.90). The highest accuracy value among the markers was observed for PIC (0.704) (Table 4).

**Table 4:** Accuracy evaluation of DD (D-dimer), PIC, SF and TAT

	Accuracy evaluation					
	Sensitivity	Specificity	PPV	NPV	Accuracy	AUC
DD (≥0.37)	0.618	0.731	0.695	0.645	0.667	0.691
PIC (≥0.80)	0.529	0.881	0.818	0.648	0.704	0.784
SF (≥0.90)	0.603	0.791	0.745	0.662	0.696	0.734
TAT (≥0.82)	0.603	0.582	0.594	0.591	0.593	0.588

Sensitivity, specificity, positive predictive value (PPV), negative predictive value (NPV) and accuracy in detecting STS is shown, according to the threshold values of 0.37, 0.80, 0.90 and 0.82

### Binary variable logistic regression analysis of DD, PIC, SF and TAT

Threshold values for DD, PIC, SF and TAT were used as binary variables in a univariate analysis. The analysis revealed significant differences in DD (≥0.37) (OR: 4.40, 95%CI: 2.12–9.09, *p* < 0.0005), PIC (≥0.80) (OR: 8.26, 95%CI: 3.44–19.9, p < 0.0005), SF (≥0.90) (OR: 5.74, 95%CI: 2.68–12.3, p < 0.0005) and TAT (≥0.82) (OR: 2.11, 95%CI: 1.06–4.20, *p* = 0.0325). These variables were utilized in further multivariate analysis with age and tumor size (Table 5). The multivariate analysis indicated that PIC (≥0.80) (OR: 3.36, 95%CI: 1.19–9.43, *p* = 0.0212) and SF (≥0.90) (OR: 2.63, 95%CI: 1.04–6.66, *p* = 0.0404) demonstrated significant differences. The values for PIC (over 0.80) and SF (over 0.90) indicated that they represent suitable markers for detecting STS (Table 5).

**Table 5:** Logistic analysis of binary variables

Univariate logistic analysis				Multiple logistic analysis			
Factor	OR	95%CI	*p*-value	Factor	OR	95%CI	*p*-value
DD (≥0.37)	4.40	2.12–9.09	**0.0005>**	Age (≥60)	1.56	0.62–3.90	0.344
PIC (≥0.80)	8.26	3.44–19.9	**0.0005>**	Tumor size (≥10)	1.94	0.84–4.42	0.116
SF (≥0.90)	5.74	2.68–12.3	**0.0005>**	DD (≥0.37)	1.94	0.73–5.18	0.179
TAT (≥0.82)	2.11	1.06–4.20	**0.0325**	PIC (≥0.80)	3.36	1.19–9.43	**0.0212**
				SF (≥0.90)	2.63	1.04–6.66	**0.0404**
				TAT (≥0.82)	0.71	0.27–1.84	0.491

DD, PIC, SF and TAT were converted to binary variables (high or low) according to their respective thresholds. Multivariate analysis was analyzed with age (≥60) and size (≥10)

## Discussion

As soft tissue tumors have various subgroups and histological variants, pathological diagnosis is a critical tool for making objective decisions on treatment strategies. Occasionally, pathological diagnosis and differentiation between benign tumors or STS is complicated. To screen carcinoma, blood tests for CEA (carcinoembryonic antigen), SCC (squamous cell carcinoma) antigen, alpha-fetoprotein, or CA-125 are useful [7]. However, blood examinations to screen for STS have not been established. Notably, the intimate relationship between coagulation and fibrinolysis in malignant tumors is a well-known phenomenon. An association between coagulation activity, INR (international normalized ratio) and cancer prognosis, such as lung cancer, colorectal cancer, pancreatic cancer, or breast cancer, has been reported [8–12]. Increased DD in plasma is associated with poor prognosis in cancer patients such as breast cancer, renal cell carcinoma, gastric cancer, lung cancer, bladder cancers, colorectal cancers, gynecological tumors and lymphoma [5, 13–17]. Among coagulation and fibrinolysis factors, DD is one of the most reported factors analyzed as a prognostic marker. However, coagulation and fibrinolysis factors are rarely reported for distinguishing between benign and malignant tumors. Previously, we reported that fibrinogen levels were an effective marker for diagnosing the differentiation of benign tumors and STS [18]. Currently, few markers are available for estimating the state of coagulation and fibrinolysis. In this study, we evaluated whether fibrinolysis markers (DD and PIC) and coagulation markers (SF and TAT) could be used to differentiate benign tumors and STS. DD is the most well-known fibrinolysis marker and has been reported as a marker capable of differentiating between benign and malignant ovarian tumors [19, 20]. In musculoskeletal tumors, DD levels showed a potential to discriminate between well-differentiated liposarcoma and lipoma [21]. Furthermore, PIC has been used for detecting breast cancer [22], and TAT was reported to distinguish between benign and malignant breast and ovarian tumors [20, 22]. Given these reports, coagulation and fibrinolysis markers may be more generally useful for distinguishing between benign and malignant tumors. However, there have been no reports investigating the accuracy of coagulation and fibrinolysis markers in detecting STS. Thus, the current study compares the accuracy of coagulation and fibrinolysis markers for detecting STS.

A comparison of plasma levels of DD, PIC, SF and TAT in benign tumors and STS indicated that DD, PIC and SF levels were significantly higher in STS. Additionally, logistic analysis using continuous variables indicated that PIC showed a significant difference, while the other factors did not. This indicates that increased PIC levels correspond to increased risk of STS. In contrast, univariate analysis of threshold values indicated that DD (≥0.37), PIC (≥0.80), SF (≥0.90) and TAT (≥0.82) showed significant differences. In comparison, multivariate analysis demonstrated significant differences for PIC (≥0.80) and SF (≥0.90). This indicates that populations with PIC and SF levels over 0.80 and 0.90, respectively, include many STS patients. Additionally, among the markers evaluated, PIC had a higher positive predictive value and a higher accuracy than the others. We previously reported that the AUC of fibrinogen for identifying STS was 0.805 [18]. Here, only PIC (0.784) exhibited an AUC that was roughly equivalent to fibrinogen. Thus, in order to detect STS, the PIC values over 0.80 represent the most effective marker of the four coagulation and fibrinolysis markers examined. According to previous STS studies, conversion of plasminogen to plasmin was observed on the sarcoma cell surface [23]. High expression of uPAR in tumor tissues and high levels of serum uPAR were associated with poor prognosis [24]. This indicates that fibrinolysis plays an important role in sarcoma progression. While DD generation requires both fibrin formation (coagulation) and degradation (fibrinolysis), PIC is an enzyme complex formed after plasmin generation. Thus, PIC is likely a faster and more sensitive marker of fibrinolysis activity than DD in STS patients.

In the patient population of this study, the STS group contained many elderly patients. Epidemiological studies indicate that the incidence STS increases with age [25] and an age of 52.7 years was delimited with 73.8% sensitivity and 52.3% specificity (AUC: 0.64, 95%CI 0.56–0.72) for distinguishing benign tumors and STS [26]. In this study, an age of 55 years was delimited with 58.8% sensitivity and 71.6% specificity (AUC: 0.737, 95%PI 0.653–0.821) by ROC analysis. Thus, age could be one of the references for the distinction of STS. Additionally, DD, PIC, SF and TAT exhibited higher values in patients over 60 years of age. It was thought that there is some association between age and DD, PIC, SF and TAT. In contrast, tumor size is a critical factor in predicting a patient’s prognosis in STS. The thresholds for staging STS are 5, 10 or 15 cm, according to the 8th AJCC system. In distinguishing benign soft tissue tumors and STS, a threshold of 5 cm is a weak predictor of malignancy (AUC: 0.663, sensitivity 68.8%, specificity 50.3%) [26]. In this study, a size of 9.5 cm was delimited with 58.8% sensitivity and 71.6% specificity (AUC: 0.72, 95%CI 0.636–0.805) by ROC analysis. Additionally, DD, PIC and SF showed higher values in patients with tumor sizes ≥10 cm. Some association between tumor size and DD, PIC and SF was considered as well as age. However, the results of the multivariate logistic analysis revealed that age and size were not significant indicators, and they did not contribute to detecting STS along with PIC or SF.

## Conclusion

Of the coagulation and fibrinolysis markers studied herein, increased PIC levels were related to STS and over 0.80 PIC was the most suitable for predicting STS; thus, along with other diagnostic tools, representing a helpful subsidiary tool for predicting malignant soft tissue tumors.

The limitations of this study include that it was a retrospective study with a small number of patients. Furthermore, statistical analysis could not be performed according to each subtype because soft tissue tumors, including sarcomas, have many subtypes and the incidence rate of each is low. Many studies have resorted to analyzing STS as a whole rather than by each histological classification. Soluble DD, PIC, SF, and TAT are influenced by trauma, surgical treatment, thrombosis, and disseminated intravascular coagulation (DIC). Patients with these backgrounds were not included in the statistical analysis of this study; moreover, no patients with DIC were observed by blood test.

## Data Availability

The datasets generated and analyzed during the current study are available from the corresponding author upon reasonable request.

## Abbreviations

DD: D-dimer
PIC: Plasmin-α2 plasmin inhibitor complex
SF: Soluble fibrin
TAT: Thrombin-antithrombin III complex
STS: Soft tissue sarcoma
OR: Odds ratio
RFS: Recurrence free survival
MFS: Metastasis free survival
OS: Overall survival
AUC: The area under the curve
ROC: Receiver operating characteristic
